# The Effect of Smoking Cessation on Body Weight and Other Metabolic Parameters with Focus on People with Type 2 Diabetes Mellitus

**DOI:** 10.3390/ijerph192013222

**Published:** 2022-10-14

**Authors:** Stamatina Driva, Aliki Korkontzelou, Serena Tonstad, Nikolaos Tentolouris, Paraskevi Katsaounou

**Affiliations:** 1 Diabetes Centre, First Department of Propaedeutic Internal Medicine, Medical School, Laiko General Hospital, National and Kapodistrian University of Athens, 11527 Athens, Greece; 2 Medical School, National and Kapodistrian University of Athens, 11527 Athens, Greece; 3 First Department of Critical Care, Evangelismos General Hospital, National and Kapodistrian University of Athens, 10676 Athens, Greece; 4 Department of Preventive Cardiology, Oslo University Hospital, 0424 Oslo, Norway

**Keywords:** smoking cessation, weight gain, nicotine, appetite-related peptides, diabetes, treatment for smoking cessation

## Abstract

Smokers with diabetes mellitus substantially lower their risks of microvascular and macrovascular diabetic complications, in particular cardiovascular disease, by quitting smoking. However, subsequent post-smoking-cessation weight gain may attenuate some of the beneficial effects of smoking cessation and discourage attempts to quit. Weight gain can temporarily exacerbate diabetes and deteriorate glycemic control and metabolic profile. The molecular mechanisms by which quitting smoking leads to weight gain are largely associated with the removal of nicotine’s effects on the central nervous system. This review addresses mechanisms of post-smoking-cessation weight gain, by reviewing the effects of nicotine on appetite, food intake, eating behaviour, energy expenditure, fat oxidation and appetite-regulating peptides. We also highlight correlations between post-cessation weight gain and risk of type 2 diabetes, consequences of weight gain in people with type 2 diabetes and the role of pharmacotherapies, which combine treatment of nicotine addiction and promotion of weight control.

## 1. Introduction

Post-smoking-cessation weight gain (PSCWG) remains a cause of concern and an independent predictor of failure of a cessation attempt, mainly among teenage girls and women [1,2,3,4,5]. Moreover, the belief that smoking can be a weight-control strategy and fear of PSCWG have been reported as reasons to initiate or continue smoking or relapse after initiating smoking cessation [6,7]. Among smokers with type 2 diabetes mellitus (T2DM), PSCWG has been associated with cessation difficulties. Two-thirds of patients with diabetes claim that they would return to smoking if they gained substantial weight [8]. Given the magnitude of risks associated with smoking, particularly in persons with T2DM, a better understanding of how to approach the problem is needed. Thus, this review covers the amount of weight gain after cessation, mechanisms of weight gain, particularly in regard to the effects of nicotine and smoking cessation on appetite-regulating peptides, health risks of PSCWG in diabetes and the effects of anti-smoking medications on weight gain.

## 2. Smoking Cessation and Body Weight in Numbers

### 2.1. PSCWG in General Population

Smoking and body weight have been correlated in several studies. Adult smokers weigh, on average, 4–5 kg less than non-smokers [9,10]. Compared to non-smokers, they are also less likely to be overweight or obese and tend to gain weight when they quit smoking [11]. Quitting smoking is associated with absolute weight gain and the differences between quitters and those who continue smoking range from 2.6 to 5.3 kg, reflecting a significant variability in the amount of weight gain [12]. 

Former smokers weigh more than both smokers or non-smokers [13]. Quitters tend to increase their body weight by a mean of 2.8 kg among men and 3.8 kg among women and over 10% of people who stop smoking gain 13 kg or more [10]. According to Lycett et al., people who successfully quit smoking typically gain an average of 8.79 kg within eight years of quitting, while continuing smokers gain 2.24 kg during the same time, a difference of 6.55 kg. However, 42% of people gain over 10 kg [14]. Aubin et al. conducted a meta-analysis including 62 smoking cessation clinical trials, which showed that smoking cessation is associated with a mean weight gain of 4–5 kg after 12 months of abstinence, and most of the weight gain occurs in the first 3 months of quitting, after which the rate of increase declines. They also noted that, within 12 months, 16–21% of participants lost weight, whereas 13–14% gained more than 10 kg [15]. In a prospective community-based cohort study using data from the Framingham Offspring Study collected from 1984 to 2011, it was observed that PSCWG was most prominent in recent (≤4 years) quitters but decreased thereafter [16]. Using data from 35 population-based prospective cohort studies worldwide, Tian and colleagues found that people who quit smoking gained an average of 4.1 kg over 5 years, compared to 1.5 kg of weight gain for continuing smokers [17]. A study of 12.204 adults from the National Health and Nutrition Examination Survey examined PSCWG over 10 years and concluded that, at 10 years, active smokers gained 3.5 kg versus 8.4 kg for abstinent smokers [18]. More recently, among 10.087 eligible smokers in the Nurses’ Health Study and 9.271 in the Nurses’ Health Study II, the estimated 10-year average PSCWG was between 3.4 and 7.9 kg [19]. 

### 2.2. The Amount of PSCWG in People with Diabetes

Among smokers with diabetes mellitus (DM), most of the existing literature on the amount of PSCWG was extrapolated from general populations. In a cross-sectional study of smokers with and without DM who enrolled in a state quitline, it was observed that participants with DM reported more weight gain in previous quit attempts than those without DM, and this weight gain appeared to be attributable to the increased prevalence of obesity and depression in these patients [8]. Clair et al. reported that similar to people without DM, among people with DM, recent quitters gained significantly more weight than smokers, long-term quitters and non-smokers [16]. A retrospective cohort study of adult smokers with T2DM, using a large U.K. primary care database, found that the mean weight gain was 5 kg in the first year after smoking cessation [20]. In two large prospective cohort studies among U.S. men and women with T2DM, the median PSCWG within 6 years was 3.2 kg [21]. Tonstad et al. concluded that mean body weight change from baseline to week 12 in quitters with DM (1.7 kg) was similar to that of those without DM (2.1 kg), in data extracted from 15 double-blind, randomized, placebo-controlled studies [22]. A more recent study suggested that among individuals with obesity and T2DM weight loss outcomes at 1 year after participating in a weight loss intervention program were not affected by smoking status [23].

### 2.3. Factors That Affect PSCWG

There is significant heterogeneity in the magnitude of weight gain following smoking cessation. Smokers who are underweight (body mass index (BMI) < 18.5 kg/m^2^), overweight (BMI 25–29.0 kg/m^2^) or obese (BMI ≥ 30 kg/m^2^) [14] tend to have the greatest PSCWG [18,24]. Additionally, those who initiate smoking earlier may also gain more weight [14]. Individuals with a high Fagerström Test for Nicotine Dependence (FTND) score are more likely to gain weight during smoking cessation therapy [25,26]. Heavy smokers (>15 or 25 cigarettes per day) have greater body weight, higher BMI and an increased risk of becoming obese compared to light smokers [7,27], and those with the highest daily cigarette consumption gain more weight after smoking cessation [18,25,26]. Smokers who report minimal physical activity and increased appetite are also at increased risk of PSCWG [25]. Gender and age-related differences in PSCWG have also been studied. Some studies show greater weight gain in men and others higher weight gain in women [24,28]. Others have found that younger smokers gain more weight than older ones [29]; however, opposite results have also been reported [28]. Pancova et al. concluded that excess weight gain in the first few months after quitting smoking is a poor predictor of overall excessive weight gain. Furthermore, lower baseline BMI, female sex, starting smoking in adulthood and higher post-cessation appetite ratings were independently associated with greater weight gain at 1 year [30]. 

## 3. Mechanisms of How Smoking Cessation Leads to Weight Gain

**Figure 1:**
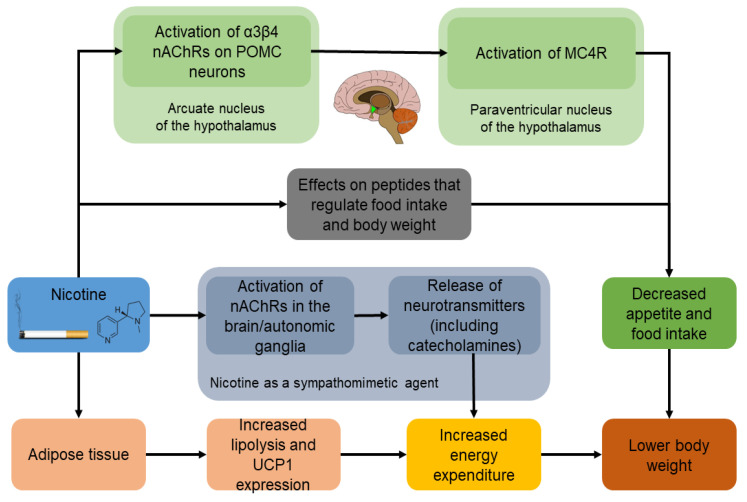
Mechanisms by which nicotine reduces body weight. Nicotine activates α3β4 nAChRs on POMC neurons in the arcuate nucleus, which then project to the paraventricular nucleus of the hypothalamus, where they activate the MC4R, suppressing appetite and inhibiting food intake. The effects of nicotine on appetite are also attributed to complex effects on peptides that regulate food intake and body weight. Nicotine also increases energy expenditure by sympathomimetic actions and through the regulation of brain metabolism. Finally, nicotine increases thermogenesis and lipolysis and stimulates the expression of UCP1 in adipose tissue, resulting in a rise in basal metabolism [32]. nAChRs: Nicotinic acetylcholine receptors; POMC: Pro-opiomelanocortin; MC4R: melanocortin-4 receptors; UCP1: Uncoupling protein 1.

Although there is convincing evidence for the inverse association between tobacco smoking and body weight, the molecular mechanisms underlying this relationship are yet to be completely understood. The major constituent of tobacco smoke that affects body weight is nicotine. Nicotine reduces body weight by suppressing appetite and food intake, increasing energy expenditure, raising the resting metabolic rate and increasing lipolysis and fat oxidation [9]. Weight gain associated with smoking cessation is largely due to the removal of the effects of nicotine on the central nervous system [9,12,31,32]. Some of these effects are presented in Figure 1.

### 3.1. Effect of Nicotine on Appetite and Food Intake

Nicotinic acetylcholine receptor (nAChR) subtypes α3β4, α7 and α4β2 have been implicated in the regulation of body weight by nicotine [33]. NAChRs are widely expressed throughout the central nervous system and periphery, including regions of the hypothalamus and, specifically, the arcuate nucleus of the hypothalamus [34]. Nicotine acts on the lateral hypothalamic area to suppress appetite and decrease food intake [34]. Geha et al. found that smokers had a greater response to milkshakes in the hypothalamus, compared with non-smokers, suggesting that smoking may influence the response of the brain to food intake in the hypothalamus. This area of the brain is significantly associated with the maintenance of body weight in non-smokers, as shown in functional magnetic resonance imaging (MRI) studies [35]. The pro-opiomelanocortin (POMC) neurons are among the best-known appetite-inhibiting cells in the mammalian brain and their activation is one of the main mechanisms by which nicotine decreases food intake [33]. Nicotine binds to α3β4 nAChRs on POMC neurons in the arcuate nucleus, which then project to the paraventricular nucleus where they activate the melanocortin-4 receptors (MC4R), inhibiting food intake. Knocking down either the acetylcholine receptor on the POMC neurons or MC4R in the paraventricular nucleus significantly blocks nicotinic-induced hypophagia, confirming that these receptors are critical to feeding regulation [36].

### 3.2. Effect of Nicotine on Eating Behaviour

One of the most common and long-lasting symptoms of tobacco withdrawal is increased appetite [37], as the appetite-suppressant effects of nicotine on the brain are reversed, which results in increased hunger and increased food intake after smoking cessation [9,38]. Increased consumption of foods high in fat and sugar activates the reward system in the brain in a similar way to smoking [39]. Low satiety, emotional eating and short sleep might also contribute to PSCWG. Some quitters also attempt to substitute the ‘hand to mouth’ behavior of smoking with eating, which can lead to an increase in caloric intake [12]. 

### 3.3. Effect of Nicotine on Energy Expenditure

As nicotine binds to the nAChRs in the brain and autonomic ganglia, ion channels open, allowing entry of sodium and calcium followed by the release of various neurotransmitters such as dopamine, norepinephrine, serotonin and other neurotransmitters in the central nervous system, including the systemic release of catecholamines [40,41,42]. Therefore, nicotine increases energy expenditure through sympathomimetic mechanisms and through the regulation of metabolism in the brain [9]. Smoking increases 24 h energy expenditure by approximately 10% [43]. This increase is more pronounced during exercise and after eating than at rest [44]. A 10% increase in 24 h energy expenditure corresponds to approximately 200 kcal per 24 h, and under the condition of a stable caloric intake, this change may result in the loss of 10 kg of body weight over 1 year [9]. The variability in the mean resting metabolic rate reduction after smoking cessation ranges from 4% to 16% and is responsible for less than 40% of the PSCWG [31].

### 3.4. Effect of Nicotine on Lipolysis and Fat Oxidation

Fat oxidation in smokers increases with increasing nicotine uptake, an observation that might contribute to PSCWG [45]. Nicotine increases thermogenesis in adipose tissue by increasing lipolysis and recycling fatty acids into triglycerides [46]. In addition, the activation of nicotine receptors stimulates the expression of uncoupling protein 1 (UCP1) within white and brown adipocytes, which results in a rise in basal metabolism [47]. The increased body weight after smoking cessation could be attributed to increased body fat based on observations that waist circumferences of quitters are higher than those of continuing smokers [48]. After 3 months of non-smoking, body weight and fat mass have been observed to increase by 4 and 22% respectively, while the percentage of lean body mass decreased by 5% [49]. Although smoking cessation is associated with weight gain mainly through the accumulation of extra fat, concomitant increases in muscle mass, muscle strength and bone density have also been observed [50]. 

### 3.5. Inverse Results of Smoking in the Long-Term

Notably, after many years of smoking, the effects of smoking on suppressing appetite and energy intake may diminish, possibly due to aging [7]. While nicotine acutely activates systems that suppress appetite and increase energy expenditure, long-term effects may reflect activation of systems that increase appetite and decrease metabolic rate [38]. Therefore, these observations could partly explain the observation that, despite their smoking, heavy smokers have a higher mean BMI and an increased risk of becoming obese, compared to light smokers [7]. A positive correlation has been observed between the number of cigarettes smoked and central fat accumulation [51]. The higher risk of obesity in smokers with high amounts of cigarette consumption may also be explained by the tendency of these smokers to adopt behaviors favoring weight gain (low physical activity, unhealthy diet, high alcohol intake) [27].

## 4. Effects of Nicotine and Smoking Cessation on Appetite-Regulating Peptides

The suppression of appetite by nicotine has been attributed to various and complex effects of nicotine on peptides that regulate food intake and body weight, such as hypothalamic neuropeptides (neuropeptide Y, orexins), adipokines (leptin, adiponectin) and other metabolic hormones (ghrelin, GLP-1) [38,52]. We focus on studies conducted in humans and mention only a few animal studies. The latter involve peptides, which have not received much attention in regard to smoking or smoking cessation in humans. We also present associations between smoking status, levels of peptides, appetite and body weight. A summary of the literature we identified is presented in Table 1.

### 4.1. NPY

Neuropeptide Y (NPY), a peptide that increases food intake, is classified as part of the pancreatic polypeptide family. This peptide shares 70% of its sequence homology with peptide YY (PYY) and it is located in the arcuate nucleus of the hypothalamus [33]. Some studies carried out in animals support a suppression of NPY expression, induced by nicotine administration [53,54,55]. Other laboratories reported an increase in NPY mRNA and its peptide after chronic nicotine treatment [56]. It was also suggested that chronic exposure to nicotine upregulates the NPY expression in forebrain areas involved in the regulation of feeding [56] with a simultaneous down-regulation of the NPY receptors [57]. This reduced hypothalamic NPY receptor density in response to nicotine could explain the decrease in food intake in smokers [58]. Studies that have measured plasma NPY levels in humans found these levels to be either elevated [59] in previously non-smoking adults after smoking or unchanged [60] in habitual smokers after acute smoking. Hussain at al. conducted the first study to show a relationship between cigarette smoking, NPY and body weight in humans. Smokers had decreased body weight and lower levels of NPY and leptin compared to never-smokers, whereas quitters had significantly increased body weight and higher levels of NPY and leptin compared to smokers. NPY levels were significantly correlated with body weight, BMI and waist circumference. NPY levels remained, in contrast to leptin, significantly lower in smokers and higher in quitters, even after adjusting for anthropometric parameters [61]. These results imply that the weight modulating effects of cigarette smoke directly involve effects on NPY expression independently of leptin [61]. Later, Stadler et al. reported that fasting NPY levels increased 3 months after smoking cessation [49].

### 4.2. Orexins

Orexin-A and Orexin-B are neuropeptides derived from prepro-orexin/hypocretin, the production of which and subsequent cleavage into orexins A and B are almost exclusively located in the area of lateral hypothalamus [57]. Similar to NPY, orexins are positive regulators of food intake. Therefore, it could be expected that orexin levels would decrease upon nicotine administration. Paradoxically, a dose-dependent increase in prepro-orexin mRNA production upon chronic nicotine administration was reported [62]. In a prospective cohort study where 200 quitters and 85 non-smokers were compared over the course of 4 weeks, a decline in orexin levels was reported during the initial withdrawal period (defined as 24 h of abstinence) among smokers who relapsed in the first 4 weeks [63].

### 4.3. Leptin

Leptin is an adipose-tissue-derived hormone that regulates both satiety and thermogenesis. Leptin suppresses food intake by decreasing appetite after eating and increasing metabolic rate [64]. It is released from adipocytes in direct proportion to fat mass [65]. There are conflicting results among different studies that examined the relation between smoking or smoking cessation and plasma leptin levels. It has been suggested that smoking, via nicotine mechanisms, might modify the sensitivity of hypothalamic leptin receptors, modulate leptin biosynthesis and consequently reduce body weight by augmenting the effects of leptin in the brain and by enhancing leptin binding or increasing the sensitivity of downstream transduction cascades [38,66]. Epidemiological studies [61,66] in different ethnic groups showed that plasma leptin, sOb-R leptin receptor and free leptin were significantly lower in smokers (moderate or heavy, cigarettes or sheesha) than non-smokers [65,67,68]. Other studies showed elevated plasma leptin concentrations in smokers [69,70] and suggested that this may be due to an increase in adipose tissue secretion of leptin or a decrease in leptin clearance [71]. In a recent study, female smokers not only showed significantly higher serum levels of leptin compared to female non-smokers, but also compared to male smokers [72]. However, most of the studies noted a significant increase in serum leptin levels after smoking cessation [69,73,74,75]. This increase was positively correlated with an increase in body weight, BMI and body fat mass [61,64,71,73,74]. Gonseth et al. reported a significant increase in serum leptin levels one year after smoking cessation, parallel with substantial weight in the same time period. This finding is in discordance with the theoretical effects of leptin. One explanation may be the development of leptin resistance in smokers [76]. More recently, Komiyama et al. found no significant changes in leptin levels in individuals with no increase in waist circumference after smoking cessation, but a significant increase in leptin levels in individuals with abdominal obesity. These observations may indicate that abdominal obesity caused leptin resistance despite smoking cessation [77]. However, another publication did not find an increase in leptin levels following smoking cessation, despite the weight gain [49]. One of the reasons for such a discrepancy might be the interaction between smoking and other factors such as diet, exercise, hormones and host inflammatory responses, which may also impair regulation of the actions of leptin [68].

### 4.4. Adiponectin

Adiponectin is a hormonal active adipocytokine secreted by adipose tissue, with insulin-sensitizing, anti-inflammatory and anti-atherogenic properties [65]. Oxidative stress and inflammatory cytokines produced by smoking, lipolysis induced by nicotine and increased consumption of circulating adiponectin, which accumulates in the injured vascular walls of smokers, are some of the mechanisms proposed to contribute to lower levels of adiponectin in smokers compared to non-smokers [46,71,78,79]. Smoking cessation improves the adiponectin profile in different time periods following cessation [80]. It has been reported in many studies that plasma concentrations of adiponectin were significantly lower in active smokers compared to non-smokers [81,82] and quitters [71,80,83,84] among both healthy persons and patients with coronary heart disease (CHD) [85]. In addition, a dose-dependent association between smoking intensity and adiponectin levels in current smokers has been observed [82]. Bergmann and Siekmeier found that in non-obese middle-aged women even moderate cigarette smoking adversely influenced the serum concentration of adiponectin, whereas among obese women there were no significant differences in adiponectin and leptin concentrations between smokers and non-smokers [65]. Efstathiou et al. reported that post-cessation adiponectin levels were significantly increased after two months in a healthy Greek population [86]. Otsuka et al. reported that plasma adiponectin levels in Japanese patients were elevated 6 months after smoking cessation [87]. In a study where early effects of smoking cessation were followed, serum adiponectin levels tended to increase 1 week after the end of treatment, but after 9 weeks, they were significantly decreased in weight gainers. In weight maintainers, adiponectin levels increased slightly after smoking cessation, but changes were not significant [88]. More recently, Komiyama et al. reported that serum adiponectin levels did not decrease 1 year after smoking cessation, despite weight gain, and increased abdominal obesity. However, in individuals with less abdominal obesity and a smaller increase in waist circumference, total adiponectin levels increased 1 year after cessation, showing the apparent beneficial effect of smoking cessation on adiponectin levels [77].

### 4.5. Ghrelin

Ghrelin is a known appetite-stimulating hormone synthesized and secreted in the stomach and hypothalamus [33]. Studies have showed that smoking acutely increased plasma levels of ghrelin, an unexpected finding in terms of explaining the known anorectic effect of smoking [89], as well as the observation that plasma ghrelin levels decreased after smoking cessation [74]. In another study, plasma concentrations of acetylated ghrelin, but not total ghrelin, were significantly higher in smokers than non-smokers [90]. More recently, in a study with a large sample size, total ghrelin serum levels were positively associated with active smoking [91]. Earlier, Kokkinos et al. found that fasting plasma ghrelin concentrations were not different between male smokers and non-smokers. Furthermore, smoking did not provoke any short-term change in ghrelin levels in smokers, but it induced a decline in non-smokers. Thus, it was suggested that if the anorectic effect of smoking is ghrelin-induced, this effect may be present only in people not habituated to smoke exposure. In habitual smokers, desensitization of receptors due to prolonged nicotine exposure could blunt ghrelin suppression by short-term smoking [92]. The lack of effect of acute smoking on serum ghrelin levels in smokers in a recent study was in line with this observation and, similarly, smoking interventions do not appear to affect levels of obestatin, which is a hormone encoded from the same gene as ghrelin [7]. These observations were not in line with previous ones, in which an acute increase in obestatin levels in smokers taking a smoke had been reported [93]. Pilhatsch et al. measured both appetite and ghrelin and found no effect of nicotine administration on ghrelin levels in healthy non-smokers, despite the decreased subjective appetite induced by nicotine. Taken together, these findings suggest that ghrelin may not play a major role in nicotine-related energy homeostasis [94]. In 26 healthy normal-weight never-smokers, Kroemer et al. found that nicotine administration decreased correlations with ghrelin levels in the mesocorticolimbic system, while subjects fasted. However, caloric load increased the modulatory effects of ghrelin on food-cue reactivity, particularly in the ventromedial prefrontal cortex and the amygdala. This effect was stronger during nicotine sessions [95]. There are also studies that showed alterations in ghrelin levels after nicotine restriction, but focused primarily on the predictive value of ghrelin measurements on smoking relapse and are outside the scope of this review [52].

### 4.6. PYY, GLP-1 and CCK

Enteroendocrine-cell (EEC) secretory hormones include PYY, glucagon-like peptide-1 (GLP-1) and cholecystokinin (CCK). A few studies have demonstrated the effect of smoking cessation on these hormones in humans [33]. PYY is an anorexigenic 36 amino-acid peptide, which is secreted by the enteroendocrine cells of the distal gastrointestinal tract. GLP-1 is a peptide produced and released postprandially by the intestinal enteroendocrine cells that acts on the hypothalamus to decrease food intake. CCK is a peptide released by enteroendocrine cells within the small intestine in response to mixed meals, acting as a satiety signal [96]. Fasting GLP-1, visfatin and PYY levels were unchanged 3 months after smoking cessation in a study conducted by Stadler et al. [49], and levels of gastric inhibitory polypeptide (GIP), GLP-1, amylin, insulin, PYY and pancreatic polypeptide (PP), measured after a meal challenge, were also not affected after 3 months of abstinence, according to Pancova et al. However, changes in incretin levels earlier after smoking cessation may have occurred [73]. PYY suppresses food intake, and although no significant change was observed on its levels after smoking abstinence, according to the above-mentioned studies, body weight showed a significant increase. More recently, Yanakkoulia et al. tried to explain the observed acute decrease in energy intake associated with acute smoking by investigating a series of hormonal factors; however, they failed to observe any significant difference in the changes in blood concentrations of CCK, GLP-1, ghrelin and obestatin over time between smoking and control condition [7]. 

## 5. Smoking Cessation and Weight Gain on T2DM and Metabolic Profile

Given what is known about the molecular mechanisms mediated by nicotine administration and smoking cessation on body weight, with focus on metabolic hormones, we now attempt to describe the connections between body weight changes and T2DM, which is known as a chronic disease strongly related with body weight condition. What explanations do we have for the relation between PSCWG and newly diagnosed T2DM, and what mechanisms are in play when smokers with T2DM quit smoking?

### 5.1. Cigarette Smoking and Risk of Diabetes

It is estimated that at least 25 million cases of T2DM worldwide may be directly attributable to cigarette smoking alone [97]. Insulin resistance, glucose intolerance, T2DM and metabolic syndrome are disturbances with strong interrelations and a common origin [27]. A number of studies have reported that smoking impairs glucose tolerance and insulin sensitivity, and increases the risk of metabolic syndrome, T2DM [27,31,68,98,99,100,101] and atherosclerotic cardiovascular disease (CVD) [102]. It is suggested that smoking, despite reducing body weight, is associated with the promotion of central obesity, hypercortisolaemia, increased levels of inflammatory markers, oxidative stress, impairment of beta cell function [103,104] and beta cell apoptosis [105]. Moreover, decreased adiponectin levels, caused by smoking, have been consistently associated with T2DM incidence [106]. Furthermore, adiponectin may play a significant mediating role in the smoking and diabetes association [68]. Smokers are 30–44% more likely to develop T2DM than non-smokers. Meta-analysis of prospective cohort studies has shown dose-dependent associations between cumulative exposure to cigarette smoke over time and incident of T2DM [97,99]. 

### 5.2. The Effect of Smoking Cessation and PSCWG on Risk of Diabetes

Insulin sensitivity seems to improve and the risk of diabetes gradually decreases after smoking cessation. Both parameters return to baseline after a few years of cessation [27]. Furthermore, a significant decrease in glycated hemoglobin (HbA1c) levels after smoking cessation has been reported [26,73]. Despite the association of smoking cessation with weight gain, concomitant improvements in insulin sensitivity and glucose homeostasis have been reported at the same time [31,107]. 

However, although smoking cessation generally tends to decrease the incidence of newly diagnosed T2DM, some studies have shown temporary increases in risk [49,88,97,108,109,110,111,112,113]. It has been speculated that PSCWG may worsen glucose tolerance and be a part of the increased risk of diabetes. Weight gain and the increase in waist circumference that occurs after quitting may influence the development of insulin resistance and attenuate the reduced risk of T2DM after smoking cessation [101,114]. In a prospective cohort study, the hazard ratio for T2DM after smoking cessation reached its peak during the first 3 years, when the maximal weight gain typically occurs, and then gradually decreased to none at 12 years following cessation [108]. Among the short-term changes in diabetes risk factors that may be observed in quitters are increases in visceral fat accumulation and waist circumference and sustained elevations of subclinical inflammation. In addition, the worsening of glucose tolerance seems to be proportional to the extent of weight gain [108,109,110]. 

The homeostasis model assessment for insulin resistance (HOMA-IR), which is used to evaluate insulin resistance and is calculated as fasting immunoreactive insulin (μu/mL) × fasting plasma glucose (mg/dL)/405, has been found significantly increased in weight gainers after smoking cessation, compared to weight maintainers [88]. Stadler et al. also found that, after 3 months of smoking abstinence, β-cell secretion in response to glucose, fasting insulin and C-peptide levels were significantly increased and these metabolic alterations may have contributed to the body weight gain after smoking cessation [49]. These findings suggest that the adverse effects of weight gain may counterbalance some of the beneficial effects of smoking cessation [77,88]. Given that PSCWG is a risk factor for T2DM, people who quit smoking should be counseled to limit their weight gain. Health care practitioners should not accept weight gain as an unavoidable side-effect of cessation [101]. 

Further observations indicate that the risk of newly diagnosed T2DM after smoking cessation is not always related to the extent of weight gain. Oba et al. indicated that although men and women who had recently quit smoking had an increased short-term risk of developing T2DM, the five-year weight gain was not associated with this risk. Individuals who had major risk factors for diabetes or who smoked heavily had a higher risk than their counterparts with fewer risk factors and lower smoking levels. It was also suggested that quitting smoking may unmask smoking-related pancreatic β-cell damage or dysfunction [115]. Similarly, in another study, ex-smokers had higher odds of newly diagnosed T2DM than current smokers during the first two years of abstinence, independently of the weight gain [116]. 

### 5.3. The Relation of Smoking to Glycemic Control and Complications in Persons with T2DM

In individuals with established T2DM, smoking has been shown to aggravate insulin resistance and impair glycemic control [100]. Among people with diabetes, smokers appear to have higher HbA1c levels than non-smokers. Furthermore, HbA1c levels tend to progressively increase with the number of cigarettes smoked per day [117,118]. Smokers are also more likely to experience severe hypoglycemia and have difficulties with insulin dose adjustment and diabetes control [12,119]. Cigarette smoking, regardless of the presence of DM, is strongly associated with vascular damage, endothelial dysfunction and activation of the blood-clotting cascade [120]. In combination with the harmful effects of glucose dysregulation, smoking accelerates vascular damage in people with diabetes and increases the risk of micro- and macrovascular complications [101,121]. The risk of macrovascular complications increases with the number of cigarettes consumed as well [122,123]. A systematic review and meta-analysis of prospective studies among people with diabetes found that smoking increased the risk of death by 48%, CHD by 54%, stroke by 44% and myocardial infarction by 52% [124]. Furthermore, higher rates of microvascular complications (including neuropathy and nephropathy, although not consistently retinopathy) have also been found in smokers. A recent meta-analysis of prospective studies found that smoking increased the risk of diabetic nephropathy [125] and another one concluded that continuing smoking and the duration of diabetes were the two major predictors of albuminuria in smokers with T2DM [126]. Finally, a meta-analysis of 10 prospective and 28 cross-sectional studies found that smoking may be associated with an increased risk of diabetic peripheral neuropathy, when studies of high quality with adequate adjustment of cofounders and longer follow-up were examined [127].

### 5.4. Smoking Cessation and Weight Gain for People with T2DM

Quitting smoking substantially reduces the risk of CVD in people with DM [128]. Clinical guidelines for the treatment of DM unanimously recommend smoking cessation as a key factor in reducing CVD for all patients [129]. However, some studies have showed that people with T2DM who quit smoking have poorer glycemic control than patients who continue to smoke [20,130]. As mentioned above, PSCWG is sometimes followed by an increased risk of glucose intolerance and metabolic syndrome, dissuading smokers, leading to interruptions and increasing recurrence rates [12]. Among people with T2DM, PSCWG may cause even greater concern because it can temporarily exacerbate diabetic symptoms through worsened glycemic control, lowering the motivation of smokers to remain smoke-free and resulting in a potential increase in the risk of microvascular complications, morbidity and mortality [131,132]. Therefore, weight control should be considered a key factor in diabetes management and be given close attention [129]. It is suggested that PSCWG should be limited to 5 kg to reduce the risk of cardiovascular events in smokers with diabetes, as well as keeping smoking abstinence for over four years [16]. However, some authors have reported that the deterioration of glycemic control in people with diabetes observed for about 3 years after smoking cessation was independent of PCSWG [20]. This issue requires further study.

### 5.5. Smoking Cessation and Macro- and Microvascular Diabetic Complications

Despite potential harmful effects of smoking cessation on glycemic control, quitting smoking has been proven to lower the risk of macrovascular complications, CVD and mortality among people with diabetes [12,101]. Smoking cessation is known to decrease long-term CVD risk in people with T2DM, independently from weight gain [16,133]. Large-scale studies and meta-analysis have shown that people with diabetes who quit smoking have a lower cardiovascular risk than continuing smokers [124,128,134] and that smoking cessation is associated with an approximately 30% decrease in all-cause mortality [135]. Among U.S. men and women with T2DM followed in two large prospective cohort studies, smoking cessation without weight gain was associated with a lower risk of CVD than people who continued to smoke. However, PSCWG did not attenuate the inverse relation between long-term cessation and all-cause mortality. These associations were independent of established risk factors, including duration of diabetes, BMI before diagnosis of diabetes, lifestyle, dietary factors and medication use [21]. As for microvascular complications, there are studies that showed that smoking cessation ameliorates the progression of the existing nephropathy and reduces levels of albuminuria in patients with T2DM [136,137,138].

## 6. Medications for Smoking Cessation and Weight Gain

The first-line medications used in the treatment of smoking cessation include nicotine replacement therapy (NRT), bupropion and varenicline [12,101]. It has been established that combined cognitive, behavioral approaches and personalized psychological support with pharmacological medications achieve the best possible results for successful smoking cessation [139]. Here, we focus on the effect of the main pharmacological approaches targeting the treatment of tobacco dependence on body weight. There are numerous studies concerning smoking cessation approaches and their effect on PSCWG in the general population, but few studies have focused on people with diabetes. A systematic review and meta-analysis that evaluated the effects of more intensive compared with less intensive interventions on smoking cessation in people with DM found only a few trials, with no evidence of efficacy [140]. Clinical guidelines for the treatment of DM include smoking cessation as a grade A recommendation, and if advice, encouragement and motivation are insufficient, then drug therapies should be considered early, including NRT, bupropion or varenicline [141]. The effect of the existing tobacco-cessation treatment on body weight and diabetes is presented in Table 2.

### 6.1. NRT

NRT is available in several formulations, all of which are effective for smoking cessation. NRT in the form of chewing gum, inhalers, lozenges, sprays and transdermal patches acts by replacing the nicotine delivered by cigarette smoking, decreasing the intensity of withdrawal symptoms and helping the smoker to quit [142,143]. With NRT, a nicotine-induced decrease in appetite and increase in basal metabolism are thought to be the mechanisms for weight-gain suppression [26]. Older studies suggested that there is a dose- and duration-dependent relationship between NRT and weight suppression and that higher replacement levels of nicotine may delay PSCWG the most [144,145,146,147]. A systematic review analyzed and compared different pharmacological interventions for preventing PSCWG and found that treatment with NRT attenuated PSCWG, with no strong evidence that the effect differed for the different forms of NRT. Furthermore, longer courses of NRT were not associated with reduced weight gain at 12 months and there was no significant dose dependent difference in weight gain at the end of treatment or at 12 months [148]. The cited review was updated recently and concluded that there was moderate-certainty evidence that NRT reduced weight at the end of treatment and that the effect may be similar at 12 months [149]. Another network-meta-analysis reported that nicotine patches in combination with other medications, and especially fluoxetine, had superior efficacy in controlling PSCWG than placebo treatment, and that nicotine patch 14 mg plus fluoxetine 40 mg treatment might be one of the best options for patients with obesity and nicotine dependence. In regard to the various NRT formulations, nicotine spray was associated with the best success rate and least weight gain [150]. Concerning people with DM, it has been reported that, as nicotinic substitutes have the same effects as nicotine on sympathetic nervous system and catecholamine release, they can have a negative impact on endothelium, cardiovascular system and glucose metabolism [151,152,153]. It has also been proposed that NRT in people with DM with poor glucose control may increase insulin resistance [100,151]. Therefore, it is suggested to limit the use of NRT in people with DM over time [101]. However, these concerns have not been examined in randomized controlled clinical trials and any potential risks of NRT are bound to be lower than those of continued smoking.

### 6.2. Bupropion

Bupropion is an antidepressant approved for smoking cessation, which inhibits the re-uptake of norepinephrine and dopamine in the central nervous system and acts as a non-competitive antagonist of nicotine receptors [154]. Randomized controlled trials have shown that bupropion is able to limit PSCWG [155,156]. Compared with a placebo, the mean body weight gain at the end of treatment was significantly lower in patients receiving short-term bupropion [155], long-term bupropion or bupropion plus nicotine patch [156]. Bupropion plus nicotine patch resulted in significantly less weight gain than treatment with bupropion alone [156]. A meta-analysis of randomized trials compared NRT, bupropion and varenicline on their effect on reducing weight gain among abstainers during 6 months’ follow-up. This report suggested that, of all three licensed medications, bupropion has the largest effect on weight gain. However, there is no strong evidence that bupropion reduces weight gain in the long-term [148,149]. Bupropion increases levels of dopamine and norepinephrine in the brain, similar to the effects of nicotine, providing a potential mechanism of weight control [9], decreases the nicotine-reward threshold and attenuates the effect of food reward [157]. Bupropion is safe for people with DM and, considering its ability to reduce PSCWG, it could therefore be proposed as a treatment of choice in obese patients with DM [101]. It also seems to improve glycemic control and loss of body weight when used as an antidepressant for patients with depression and DM. While the medication insert advises caution in patients using insulin or other medications that could cause hypoglycemia, due to the possibility of lowering the seizure threshold, this risk was not observed in clinical practice [158].

### 6.3. Varenicline

Varenicline is a selective partial agonist of the α4β2 nAChRs in the brain. Varenicline acts by attenuating withdrawal symptoms during a quit attempt and is more effective than the previous mentioned therapies [159]. Varenicline attaches to nicotinic receptors in the brain, releasing dopamine. This action has been suggested to contribute to the suppression of appetite [26]. Older studies showed a greater amount of weight gain in the varenicline group compared to the amount observed in smokers randomized to a placebo, NRT or bupropion during smoking cessation [148,160,161,162]. A meta-analysis conducted by Aubin et al. reported that smoking cessation with varenicline resulted in 0.4 kg of weight gain after one year of abstinence, whereas the weight gain observed with the nicotine patch was 0.5 kg [15]. Varenicline users gained significantly less weight than nicotine patch users in studies conducted on smokers living in Japan making a quit attempt. The mean weight gain from baseline to 12 months was 0.94 kg in smokers randomized to varenicline versus 2.78 kg in smokers randomized to nicotine patches [163]. A recent meta-analysis found that, after the 12-week treatment phase, varenicline was associated with 0.23 kg less weight gain than the control therapy. However, this observed modest limitation of weight gain was small, did not differ from the effect of placebo treatment, did not last long and became non-significant during non-treatment follow-up [164]. As far as people with DM are concerned, a pooled analysis of 15 double-blind, randomized, placebo-controlled studies of varenicline in smokers with DM showed that weight gain was limited to 1.7 kg in quitters in both the varenicline and placebo groups after 12 weeks and that varenicline is an effective and well-tolerated aid for smoking cessation in people with DM [22]. 

### 6.4. GLP-1R Agonists

Recently, interest has been shown in glucagon-like peptide-1 receptor (GLP-1R) agonists, a class of medication increasingly used for diabetes control, as a potential treatment for smoking cessation. In a pilot study, extended-release exenatide, a GLP-1R agonist, added to the nicotine patch, improved abstinence, reduced craving and withdrawal symptoms and decreased PSCWG among abstainers. Findings suggested that the GLP-1R agonist strategy is worthy of further research in larger studies with longer durations [165].

## 7. Conclusions

Smoking cessation leads to weight gain via various molecular mechanisms, which consist primarily of the removal of nicotine’s effects on the central nervous system and on peptides that regulate food intake and body weight. PSCWG remains a factor of concern, particularly among smokers with T2DM, as it may lead to the deterioration of glycemic control and temporarily exacerbate diabetes control. PSCWG can also be a part of the underlying mechanism for developing insulin resistance and a possible risk factor for newly diagnosed T2DM. However, this effect is temporary and weight gain after smoking cessation does not outweigh the benefits of quitting smoking on CVD risk and mortality among adults without diabetes [16] and on micro- and macrovascular complications among people with diabetes. Smoking cessation is a key factor for all patients with DM, according to clinical guidelines for the treatment of DM, and, fortunately, effective and well-tolerated pharmacotherapies are available, which combine the treatment of nicotine addiction and promotion of weight control. 

Understanding and examining further the hormonal and neural mechanisms by which smoking cessation is related to weight gain will help to identify molecular targets for new medications for smoking cessation and PSCWG treatment. Most of the studies in the international literature refer to smoking cessation in healthy participants or other population groups and not in people with DM, who constitute a special and very sensitive population group and should be constantly encouraged to quit smoking by health care professionals. Future studies are needed, which will examine mechanisms of PSCWG in people with DM, along with long-term interventions and new medications for smoking cessation, concerning their effectiveness in reducing PSCWG and focusing on smokers with T2DM.

## Figures and Tables

**Table 1:** Summary of the literature on the effect of nicotine and smoking cessation on NPY, orexins, leptin, adiponectin, ghrelin, PYY, GLP-1 and CCK.

Appetite-Regulating Peptides	Smoking	Smoking Cessation
NPY	↓ levels of NPY	↑ levels of NPY correlated with body weight
Orexins	dose-dependent ↑ prepro-orexin mRNA production upon chronic nicotine administration	↓ orexin levels during the initial withdrawal period (24 h of abstinence)
Leptin	↓ or ↑ plasma leptin concentration	↑ serum leptin levels positively correlated with ↑ body weight, BMI and body fat mass
Adiponectin	↓ plasma concentrations of adiponectin	↑ serum adiponectin levels in individuals with less abdominal obesity
Ghrelin	↑ or ↓ plasma levels of ghrelin, non-effect result in smokers	No data
PYY, GLP-1 and CCK	Not affected	Not affected

NPY: neuropeptide-Y; PYY: peptide YY; GLP-1: glucagon-like peptide-1; CCK: cholecystokinin; ↑: increased; ↓: decreased.

**Table 2:** Summary of literature on the effect of smoking-cessation treatment on body weight and diabetes.

Smoking-Cessation Treatment	Weight Change	Diabetes Effect
NRT	weight-gain suppression	can have a negative impact on glucose metabolism
Bupropion	maybe the largest effect on weight-gain suppression	safe for people with DM, treatment of choice in obese patients and patients with depression and DM
Varenicline	modest limitation of weight gain	effective and well-tolerated treatment for smokers with DM

NRT: nicotine replacement therapy; DM: diabetes mellitus.
